# Efficiency Decreases in a Laminated Solar Cell Developed for a UAV

**DOI:** 10.3390/ma15248774

**Published:** 2022-12-08

**Authors:** Krzysztof Mateja, Wojciech Skarka, Aleksandra Drygała

**Affiliations:** 1 Department of Fundamentals of Machinery Design, Silesian University of Technology, Stanisława Konarskiego 18A, 44-100 Gliwice, Poland; 2 SkyTech eLab LLC, Stanisława Konarskiego 18C, 44-100 Gliwice, Poland; 3 Department of Engineering Materials and Biomaterials, Silesian University of Technology, Stanisława Konarskiego 18A, 44-100 Gliwice, Poland

**Keywords:** renewable energy, flexible solar cell, lamination, energy harvesting, UAV power supply system

## Abstract

Achieving energy autonomy in a UAV (unmanned aerial vehicle) is an important direction for aerospace research. Long endurance flights allow for continuous observations, taking of measurements and control of selected parameters. To provide continuous flight, a UAV must be able to harvest energy externally. The most popular method to achieve this is the use of solar cells on the wings and structure of the UAV. Flexible solar cells mounted on the surface of the wings can be damaged and contaminated. To prevent these negative changes, it is necessary to apply a protective coating to the solar cells. One of the more promising methods is lamination. To properly carry out this process, some parameters have to be appropriately adjusted. The appropriate selection of temperature and feed speed in the laminator allows a PV (photovoltaic) panel to be coated with film, minimizing any defects in the structure. Covering PV panels with film reduces the performance of the solar cells. By measuring the current–voltage characteristics, data were obtained showing the change in the performance of solar cells before and after lamination. In the case of testing flexible PV panels, the efficiency decreased from 24.29 to 23.33%. This informed the selection of the appropriate number of solar cells for the UAV, considering the losses caused by the lamination process.

## 1. Introduction

External energy harvesting allows for standalone power supply systems to extend their working time and even achieve full energy autonomy [1,2,3]. PV (photovoltaic) panels allow electricity to be obtained from solar energy, and surplus energy can be stored in batteries [4,5]. The use of such systems is gaining more and more popularity in the electromobility industry in use, among others, in charging stations for electric cars, and in aviation as an element of the wings or other parts of the vehicle structure. In the case of UAVs (unmanned aerial vehicles), the operation of a solar cell under the conditions in which it will be operated should be verified [2]. Currently, UAVs are used for, amongst other functions, distributing shipments, mapping, surveillance, and monitoring of borders and crops [6,7]. The biggest research area associated with UAVs is increasing flight duration without unnecessary landing. For this purpose, systems should be developed to increase flight duration, optimize the system in terms of weight and provide functionality in all weather conditions. Obtaining external energy allows for energy autonomy; however, it is closely related to the location and time of flight [8]. The use of solar cells allows for an increase in flight duration, but it also has numerous limitations that have to be taken into account during the design of power supply systems [9,10,11].

The sun is the largest source of free energy on Earth. Solar energy is a renewable, pollution-free, sustainable, and inexhaustible resource. A solar cell is a device that converts solar energy into electricity through the photovoltaic effect. The most-used material for solar cells is silicon. Other materials used for the construction of photovoltaic cells are gallium arsenide, cadmium telluride, and copper indium gallium selenide. However, these technologies are restricted by resource scarcity. The highest efficiency is provided by GaAs solar cells, but these cost as much as ten times more than silicon-based devices [7,12,13]. Although different solar cell types on the market exist, very few are applied to UAVs due to their energy conversion efficiency, cost considerations, environmental compliance, weight, and flexibility.

Standard silicon solar cells are brittle and breakable, so this type is not suitable for UAVs [14,15]. In the case of aircraft, solar cells must be able to distort due to the numerous curvatures of the wing surfaces and also due to the stresses that occur on the UAV during flight [16,17,18]. Solar cells used in aviation and space applications should be flexible to better conform to the surface of the wings, tail, and hull [8,19,20].

Currently, solar cells convert most of the visible light spectrum and about half of the ultraviolet and infrared light spectrum to usable energy. The efficiency of a solar cell is a measure of its performance in converting sunlight into electricity [21]. The electrical properties of solar cells are determined from measured current–voltage (I–V) characteristics and power–voltage (P–V) characteristics.

The energy conversion is driven by the absorption of light (photon) energy, producing electron–hole pairs in a semiconductor and charge carrier separation. A p–n junction is used for charge carrier separation. For photovoltaic devices, reflection and transmission are typically considered loss mechanisms, as photons that are not absorbed do not generate power. To protect solar cells from external conditions, thin films that are resistant to mechanical damage are used [20]. Solar panel lamination is one of the processes crucial to ensuring a long lifespan, but it does affect the amount of light entering the device. In this work, the influence of a protective film application on optical properties was investigated via spectrophotometer. A spectrophotometer allows for the observation of changes in the range of UV, visible, and infrared light transmitted, and to analyze if within these ranges the characteristics of the tested samples are constant or variable [22].

Each protective layer on a solar cell’s surface together with the effects of ambient temperature and irradiation variations cause the parameters of PV panels to change [23,24,25,26]. To perform tests on solar cells, it is necessary not only to measure the current–voltage and power–voltage characteristics for different irradiation and temperatures of the solar cell, but also to check the solar cell structure and protective layers on its surface [27,28].

During the lamination process, the microstructure of the solar cell may experience changes [29]. Microfractures can be caused by environmental conditions such as thermal cycling and humidity. Another cause of micro-cracks are mechanical stresses. This kind of damage can be caused by choosing incorrect parameters for the lamination process, e.g., by using too much pressure. Micro-cracking causes a reduction in output power resulting in deterioration of the efficiency of the solar cell.

To strengthen solar cells and prevent mechanical damage, various protective coatings are used on their upper surface: films, resins, and composite materials. This process is also intended to improve the aerodynamics of the UAV. The coating protection extends the service life of the system. It allows for quick cleaning of PV panels and also prevents the ingress of moisture and dust into the system. The process of mounting solar cells on the wing can be divided into several types of technology [20] using the following methods:

- Adhering to an existing wing—this method is good for retrofitting an existing UAV. Aerodynamics are normally not affected as modules are extremely thin. The biggest advantage of this solution is it allows the possibility of replacing PV cells in the event of damage. Wiring between modules is time-consuming with large wings, as strings of solar cells run from root to tip. The biggest disadvantage of this solution is the sealing of the gap between two modules [20,30].
- Placed into a mold—the challenge is to fix the modules in their exact position and to ensure no resin leaks onto the front of the module. The advantage of this solution is the wiring, which is easy to arrange. The effects of PV modules on aerodynamics are largely eliminated but modules cannot be swapped in case of damage. One variation of this method is to place solar cells inside the wing structure with a transparent coating, e.g., transparent film. This technology is mainly practiced within hobby modeling circles and the production process can be seen on models that are often developed by enthusiasts, e.g., on YouTube channels. Due to the labor-intensive nature of this method and the impossibility of replacing damaged elements, it is rarely used in commercial UAVs.
- As the wing surface—lightweight solar modules need more ribs; more sturdy solar modules need fewer ribs but have more weight. The wiring arrangements are easy in this solution [31,32,33,34].

**Figure 1:**
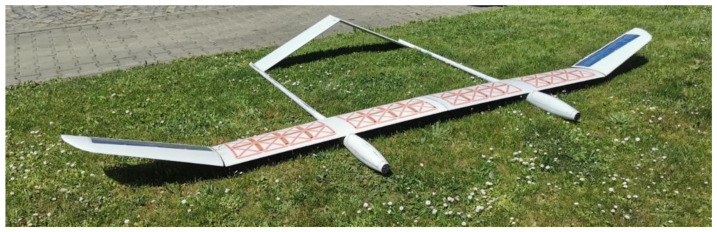
The first prototype of TwinStratos.

This article details aspects of the development of a solar-powered UAV which is designed to be able to fly in the stratosphere—TwinStratos (TS) UAV [35,36,37]. The goal of this research was to obtain an understanding of the laminated solar cells used in the first, smaller prototype of TS (Figure 1). Decreases in efficiency and changes in the parameters of solar cells can affect energy produced by the system. For the purposes of this experiment, the UAV was equipped with SunPower Maxeon Ne3 solar cells, which are flexible and allow for adaptation to curved surfaces. The manufacturer of the SunPower Maxeon Ne3 cells ensured efficiency at a level of 24.3% [38]. Data received from a test stand allowed us to calculate if the number of solar cells assumed in the initial assumption was able to perform the assumed flight mission. Data obtained in the test allowed the development of a simulation model for a power supply system of the envisioned solar-powered UAV. In previous works, this integrated design approach based on model-based system engineering developed by the project team was applied to the design and testing of ultra-efficient racing vehicles [39], automated guided vehicles (AGVs) [40], as well as for the design of general aviation class aircraft [41].

## 2. Materials and Methods

### 2.1. Lamination Process

In this study, we decided to laminate solar cells and glue PV panels to the UAV’s wings. This method of mounting was chosen due to the fact it allowed application onto an existing aircraft. The second reason was related to the proof-of-concept stage of the UAV being developed. If there were any changes needed, these could be made relatively easily.

Solar cell lamination has two purposes:

- Improving the aerodynamics of the wing with elimination of sharp edges;
- Protection against scratching of the solar cell, action of chemicals, and harmful effects of weather conditions.

A disadvantage of lamination is the reduction in efficiency of solar cells in relation to the efficiency of uncovered solar cells. The test plan relating to lamination has been divided into individual stages:

1. Testing of films of various thicknesses involving local damage to samples and then checking their reaction to external forces. This study enables the selection of a suitable film ultimately used in the UAV.
2. Examination of the selected film with a spectrophotometer to find out its characteristics of reflection, absorption, and transmission.
3. Covering the solar cell with the selected film. During lamination of the solar cells, an important aspect is the selection of appropriate process parameters.
4. Testing the current–voltage characteristics of solar cells before and after the lamination process.

### 2.2. Film for Lamination

There are a few types of film with different thicknesses that can be used as protective surfaces for solar cells. In aerospace, one of the most widely used encapsulating materials is EVA (ethylene–vinyl acetate) [42]. The advantages of this material are high transmission and resistance to UV radiation [43]. The disadvantage in the case of EVA is the method required in the lamination process. To provide a smooth connection between the film and the solar cell, it is necessary to use a vacuum, ensuring that no air or humidity will be in contact with the solar cells. This requires advanced equipment that increases the cost of making the prototype of the UAV.

Another kind of film that can be used for solar cell lamination is PVC (polyvinyl chloride) film. PVC and EVA are similar materials. EVA is more flexible, lighter, and stronger than PVC, but the advantage of PVC is its ease of application to the solar cell. In this case, use of vacuum is not necessary. The time needed to prepare PVC-laminated solar cells is shorter than in the case of EVA.

For our prototype solution, we decided to use PVC film due to the simplicity of its application to the solar cell’s surface. The films tested ranged from 60 to 250 microns in thickness. The inner side of the film is covered with glue, allowing adhesion to the laminated elements. The thinnest films were characterized by high flexibility but low mechanical strength, thick films the inverse. To select the appropriate film thickness, we decided to conduct several tests to check the strength of the films. Films were tested primarily in terms of their actual application and the typical working conditions. For this reason, at this stage of the work, no research was carried out with the use of advanced equipment, but only with the use of simple tools—knives, drills, needles, and files. Prepared damages are the most common defect that can occur during UAV flight operations. The performed tests were to show whether the damage caused by the system would allow further operation of the UAV or not. A visual method was used to check for defects appearing after film failure.

Tests have been carried out on laminated films. A laminator was used to prepare the samples. For lamination, we used a Laminator OPUS ProfiLAM (OPUS, Gliwice, Poland) A3. For the PVC film method of solar cell lamination, it was found that during the welding of the film, the guide rollers removed air just before the lamination process. With such a laminating process, there was no need to control the pressure to facilitate getting rid of air bubbles. The preparation of samples for testing began with laminating paper as a precursor to laminating solar cells. Due to its hygroscopicity, the paper allowed the adhesive to be absorbed, thanks to which no stains or air bubbles were formed. The use of paper additionally allowed us to obtain a rigid surface like the surface of a laminated solar cell. In the case of double lamination (film–paper–film), the second layer of film additionally stiffened the whole, making the sample similar to the structure of the UAV’s laminate surface.

### 2.3. Initial Film Thickness Tests

In the case of testing the mechanical strength of the film against damage in real conditions, three tests were carried out. The first test consisted of cutting the film lengthwise and then bending it. The purpose of the test was to show the reaction of the longitudinally torn film to the stress on the wing of the UAV. Defects of this type may appear in the case of incorrect performance during manufacturing. The second test consisted of creating spot damage to the film and then checking whether the defect due to bends would enlarge. The purpose of this test was to present an example of films being damaged in flight. The final test tested damage to the edges of the film and then analyzed how stresses and external forces affected this damage. This test was similar to the second test, but the film was damaged at the end of the sample. This type of damage may occur when the film is detached from the UAV structure.

Every test was conducted several times on each film type with the number of bends to the film occurring around a dozen repetitions. This number of repetitions made it possible to observe changes in the structure of the samples. In the case where changes were not noticeable, the test time and/or a change of method of loading the samples using stretching and bending along other axes were added.

**Figure 2:**
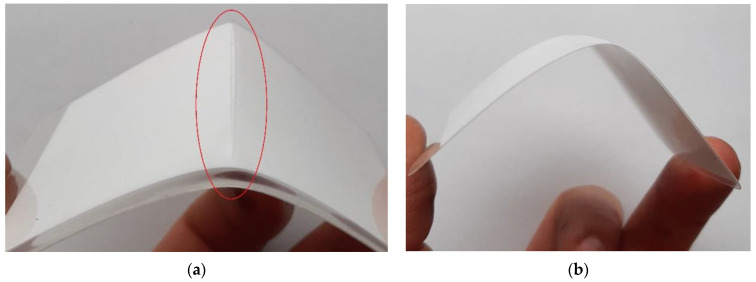
Samples tests: (**a**) incision test of a film with a thickness of 250 μm with a burst gap; (**b**) incision test of a film with a thickness of 100 μm, which does not enlarge the damage.

The incision test followed by the bend test yielded the observations in Table 1. Figure 2 presents the results of the incision test.

In the second test, related to spot damage, all film thickness did not show any enlargement of defects, even under the influence of prolonged stresses as a result of bending or applying tensile stress.

The third test, related to end damage to the film, showed the effects listed in Table 2.

Thinner films allow flexibility over a low radius equal to a few centimeters. This feature of thin films allows for their use on small UAV elements such as hulls, ailerons, and flaps. Thick films do not allow the same flexibility over a low radius as thin films do, but they are more durable. Thick films provide higher resistance to mechanical damage. However, the thicker film, the lower the efficiency of the solar cells. Thicker films are heavier than thinner films, which is another point in favor of using the thinnest possible film.

After the lamination process, solar cells are soldered. Soldered joints stiffen the PV panel, increasing its brittleness. Analyzing the research carried out on possible damage of laminated solar cells during the flight of the test UAV and its response to defects, it was decided that the thinnest film that could be used was a film with a thickness of 100 μm.

### 2.4. Parameters of the Lamination Process

While testing the film samples, the parameters of the lamination process were of less importance due to the use of absorbent paper, to which the film adhered easily. In the case of solar cells, these parameters are more important due to the non-absorptive nature of solar cells. The parameters that played the greatest role in an optimized process were temperature and speed of lamination.

**Figure 3:**
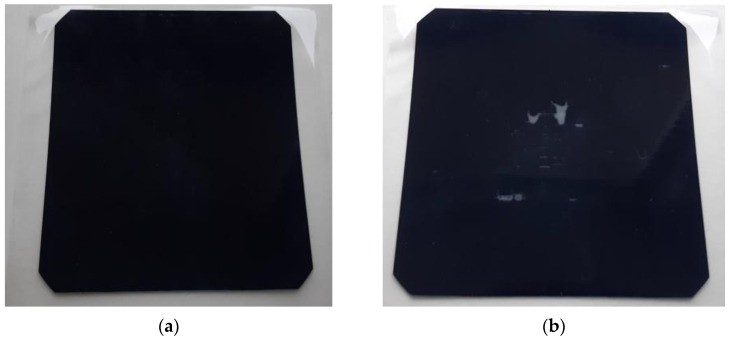

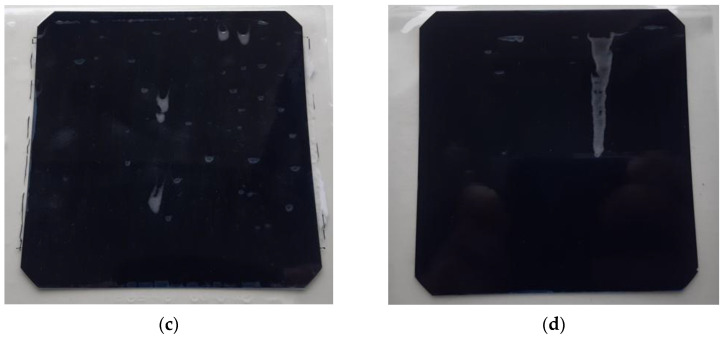
Laminated solar cells: (**a**) without defects; (**b**) adhesive stains on the surface caused by too fast feed; (**c**,**d**) damp patches from the adhesive caused by too low temperature of lamination.

An optimized lamination process should create a smooth texture on the surface of the solar cell without visible defects (Figure 3a). The laminator used allowed 9 lamination speeds, allowing a feed rate from 200 to 1800 mm/min with increments of 200 mm/min for each speed. High feed rates (lamination speed) caused the film to peel off the solar cell. A second disadvantage of high feed rates was the formation of adhesive stains on the solarcell’s surface (Figure 3b). Feed rates over 1400 mm/min produced these defects.

Another variable parameter was the temperature of lamination. The temperature range of the lamination process was between 70–140 °C. Low temperatures caused ineffective lamination, characterized by the formation of damp patches from the adhesive (Figure 3c,d).

Feed rates of 800–1000 mm/min and temperatures in the range of 90–105 °C produced optimal results.

The selection of the appropriate lamination process parameters made it possible to obtain a homogeneous PV panel surface free from flaws. Prepared samples were subjected to tests that examined their electrical properties before and after lamination.

Parallel to the lamination of the chosen solar cells, a different type of flexible solar cells was also laminated. For each type, the optimal parameters of temperature and lamination speed determined for that type were used.

## 3. Test Stands

### 3.1. Test Stand for Collecting the Characteristics of Transmission, Absorption, and Reflection

During the research, we used an Evolution 220 spectrophotometer (Thermo Fisher Scientific, Waltham, MA, USA) to measure the characteristics of transmission, absorption, and reflection of the film. The spectrophotometer allowed for the determination of the characteristics in wavelengths ranging from 190 to 1100 nm.

### 3.2. Microscale Characterization Method

To obtain images of the monocrystalline surface topography of solar cells we used a scanning electron microscope (SEM). Images were obtained using a Supra 35 (Zeiss, Thornwood, NY, USA) SEM using an acceleration voltage of 10 kV. The secondary electron (in-lens) detector was used to obtain images of the surface topography.

### 3.3. Test Stand for Collecting Current–Voltage Characteristics of a Solar Cell

**Figure 4:**
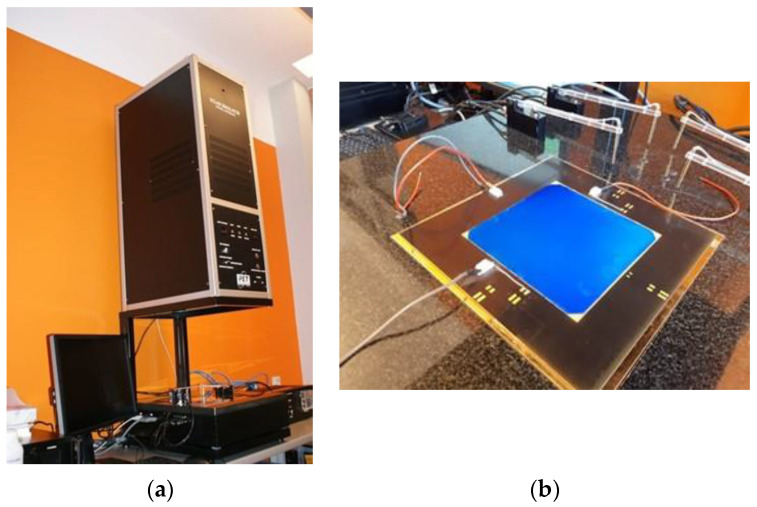
Test stand: (**a**) solar simulator with a xenon flash lamp, measuring table, and computer for downloading current–voltage characteristics; (**b**) research conducted on flexible solar cells placed on the measuring table.

The test stand (Figure 4a) for measuring the current–voltage characteristics of solar cells allowed measurements to be obtained for the tested solar cells for STC (standard test conditions)—irradiated with a power of 1000 W/m^2^ at a temperature of 25 °C, and Air mass 1.5 spectrum (AM 1.5) defined by European standard IEC 60904-3 [44]. This system for I-V characteristic measurements of solar cells meets all the requirements of the IEC 60904-1 standard [45].

The device consists of a light source in the form of a xenon flash lamp with a power of 1430 watts. After passing through the filter (“Air Mass Filter”) and the optical system, it uniformly illuminates the measuring table (Figure 4b).

## 4. Results and Discussion

### 4.1. Transmission, Absorption, and Reflection of the Film

**Figure 5:**
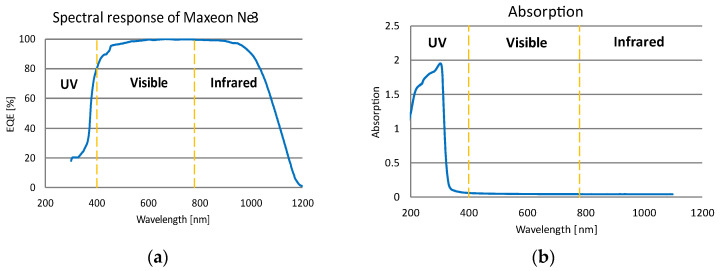

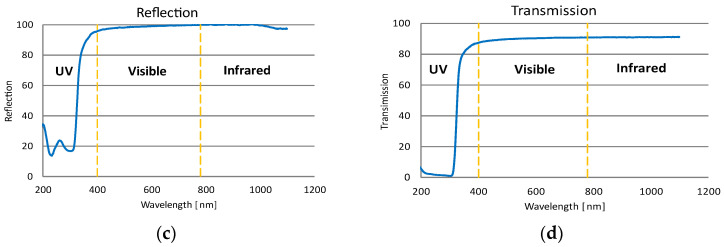
Characteristics of: (**a**) spectral response of Maxeon Ne3; (**b**) 100 μm film absorption; (**c**) 100 μm film reflection; (**d**) 100 μm film transmission.

The characteristics of absorption, reflection, and transmission are presented in Figure 5b–d for both films before and after the lamination stage were tested. The SunPower Maxeon Ne3 datasheet contains the spectral response of solar cells [38], which is presented in Figure 5a. The spectral response is the ratio of current generated by the solar cell to the power incident on the solar cell [46]. These characteristics make it possible to observe changes in the spectral response depending on the wavelength. When analyzing the graphs, the main observations were made of the wavelength range and places where the changes in characteristics occurred. In terms of the solar energy supplied to solar cells, the changes in the UV and infrared range are not as significant as in the visible light range.

There are some changes in the UV wavelength range for transmission, absorption, and reflection. From a value of about 300 nm, the characteristics stabilize at one level over the entire range of visible light up to a final value of 1100 nm. The research carried out on laminated film elucidated changes in reflection, absorption, and transmission in the visible light range. A uniform value of the characteristics in the range from 300 to 780 nm demonstrates that the parameters of the solar cell in the visible light range will be constant. This information allows the conclusion to be made that the system will operate with similar performance across the entire range of visible light.

### 4.2. Microscopic Scale Observations of the Solar Cell

**Figure 6:**
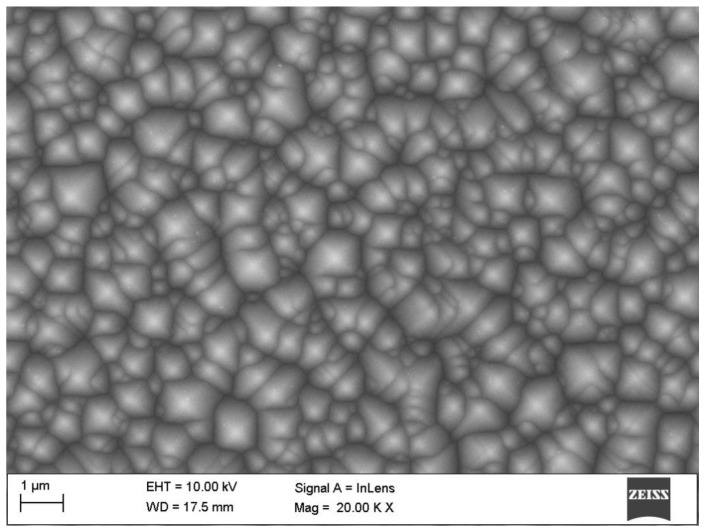
SEM textured surface topography of the N-type monocrystalline silicon solar cell.

Figure 6 shows the surface topography of a monocrystalline silicon solar cell. It was observed that there are randomly distributed pyramids on the surface, which may indicate the etching of the substrate in alkaline solutions. This chemical treatment of monocrystalline silicon significantly reduces the reflectance from the front surface of the solar cells. The texturization of the silicon surface is a key element in the production of photovoltaic cells, enabling the formation of an appropriate microstructure on the surface of the substrate, trapping solar radiation inside the material by repeated reflection [47,48,49].

**Figure 7:**
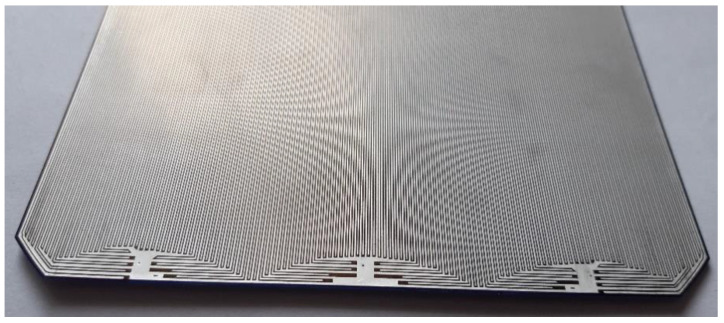
Rear surface of Maxeon Ne3 with visible connectors in the lower part.

**Figure 8:**
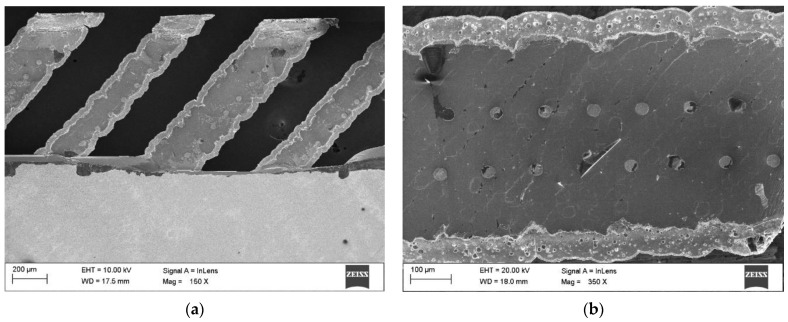
Topography of the electric contact surfaces of a monocrystalline silicon photovoltaic cell of the N-type: (**a**) contact of fingers (grid lines) with connectors; (**b**) finger (grid line).

All leads in the tested N-type solar cells are on the rear surface of the samples (Figure 7). The electrode topography of a monocrystalline silicon photovoltaic cell is shown in Figure 8a,b.

When analyzing the structure of the solar cell before and after the lamination process, no traces of microcracks were observed. The temperature changes during lamination and the force generated by rollers pressing the film to the solar cell did not damage the upper surface layer and the electrical connections of the solar cell. The decrease in the efficiency of the solar cell is therefore not due to microcracks, but only due to the properties of the layer of film applied during lamination. The lower efficiency and deterioration of electrical parameters are related to the light transmittance factor of the film.

### 4.3. Solar Cell Characteristics

**Figure 9:**
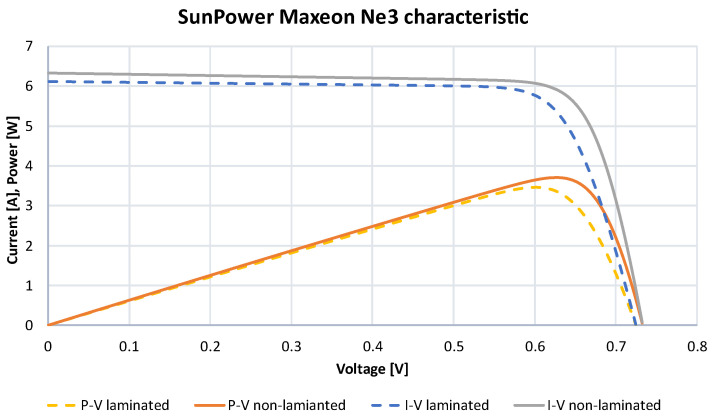
I–V and P–V characteristics of the SunPower Maxeon Ne3 cells.

The test stand allowed the determination of the electrical specification of the solar cells (Table 3). These values, as a mean of all measurements, I–V (current–voltage) and P–V (power–voltage) characteristics are presented in Figure 9.

For each type (laminated and non-laminated) we used 25 samples of solar cells to conduct research. The relative standard deviation (RSD) as well as the minimum and maximum values obtained during tests are presented in Table 4.

**Figure 10:**
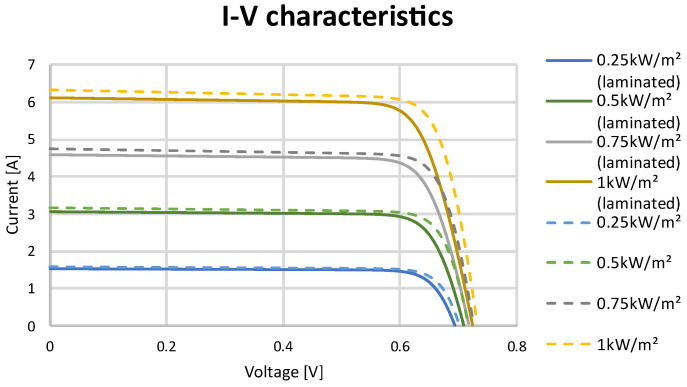
I–V curve at different irradiation levels for SunPower Maxeon Ne3 cells.

**Figure 11:**
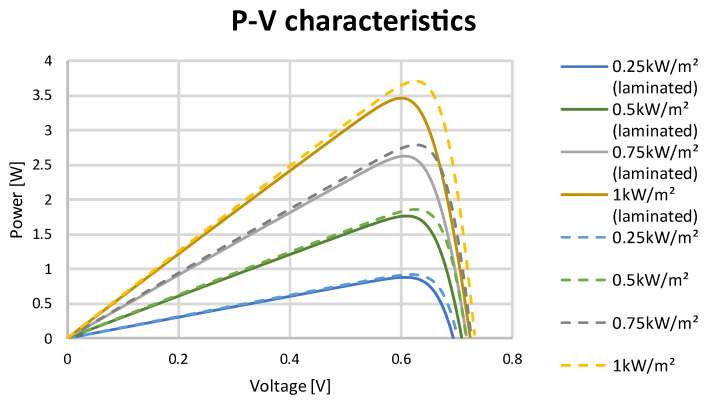
P–V curve at different irradiation levels for SunPower Maxeon Ne3 cells.

The test stand allowed for an irradiation intensity with a power equal to 1000 W/m^2^ to be provided to cells. To obtain the characteristics for the lower range of irradiation intensity, the commonly used generic simulation model using a MATLAB/Simulink system was applied. Data obtained during the STC tests of the solar cell were used as inputs in the simulation model. Data from the tested solar cells and different irradiation levels are presented in Table 5. I–V and P–V characteristics are presented in Figure 10 and Figure 11.

**Figure 12:**
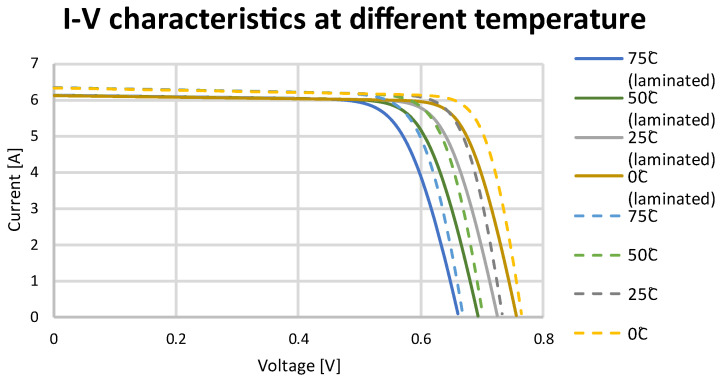
I–V curve at different temperatures for a SunPower Maxeon Ne3 cell.

**Figure 13:**
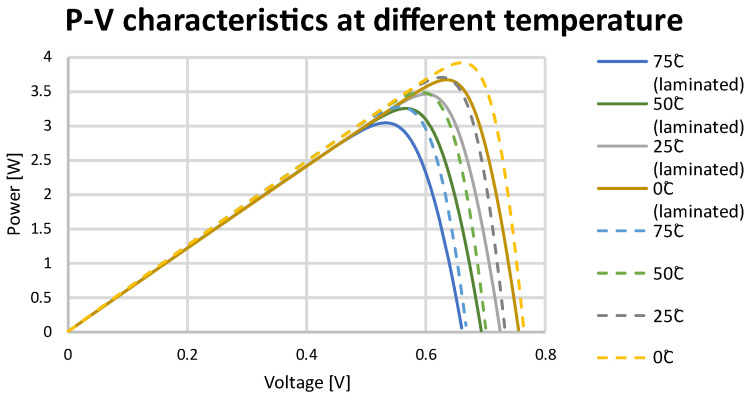
P–V curve at different temperatures for a SunPower Maxeon Ne3 cell.

The data introduced into the system allowed for the determination of current–voltage (Figure 12) and power–voltage (Figure 13) characteristics of solar cells for different temperatures. Using temperature coefficients of the SunPower Maxeon Ne3 cells, the following values were applied: voltage: −1.74 mV/°C, current: 2.9 mA/°C, power: −0.29%/°C.

Temperature coefficient data obtained in the simulation model allowed for the comparison of these data with analytical calculations. Comparing these values, it can be concluded that the simulation model results are consistent with the calculations. The data of a solar cell for different temperatures are presented in Table 6.

## 5. Conclusions

SunPower Maxeon Ne3 solar cells were selected for testing present repeatable electrical and physical parameters. The flexibility of the solar cell allows it to be bent and influenced by external forces without fear that the solar cell will be broken or damaged. These features of the cell make it suitable for aerospace applications.

Flexible solar cells were covered with a thin film to provide protection and enhancement of the solar cell and to improve the aerodynamics of the UAV structure. The reduction in the solar cells’ efficiency because of the lamination process reduced the energy supplied to the UAV power supply system. Tests carried out on test stands allowed for the determination of the efficiency of laminated and non-laminated solar cells. It was found that the optimal film thickness for lamination, a PVC film of 100 μm, reduced efficiency by 4%. Spectrophotometric characteristics of transmission, absorption, and reflection allowed the conclusion that in the full range of visible light, these values are constant. This data demonstrates that losses of efficiency are constant for the visible light range. Reductions in efficiency precipitate the need to use more solar cells to obtain the same energy value. Reduced efficiency in relation to non-laminated solar cells, together with the benefits of enhanced protection of cells that film lamination confers, result in the need to change the design of the UAV, to optimize the energy consumption or redefine the battery capacity.

Simulations that include the different parameters of solar cells in different temperatures allow for the determination of the response of the system in conditions when solar cells are exposed to frosty surroundings and to high temperatures. In the case of a specific UAV being designed as part of this work, with an optimal flight time of over 24 h, this reduction in efficiency due to lamination equal to 4% will be significant.

Further work is planned to validate the simulation models of the laminated PV panels by testing the UAV in a real environment. The simulation model will allow predictions for the control of the energy balance of the UAV. Data related to solar cells, such as sun exposure, cloud cover, location, day duration, angular variation of the aircraft, flight scenarios, and energy consumption will also be considered. These data, in combination with the development of a power supply system, will allow for the calculation of the energy balance and planning of optimal flight paths in the stratosphere.

The methods developed for lamination of solar cells and data obtained will be used in the first prototype of the TS UAV. Subsequent improved iterations of TS will be able to fly in the stratosphere and achieve a cruising altitude of 20 km. These extreme conditions will allow verification of the initial assumptions with regard to the laminated PV systems meeting the requirements of this demanding environment.

## Figures and Tables

**Table 1:** Incision test results.

Film Thickness (μm)	Effect
≤100	The incision damaged the inner side of the film. Due to the high flexibility of the film, the incision did not enlarge.
125–200	The incision damaged the inside of the film, enlarging the gap due to prolonged bending.
≥250	The incision did not damage the inner side of the film. However, due to bending, the gap burst.

**Table 2:** End damage results.

Film Thickness (μm)	Effect
<100	In the case of thin film, the defect easily increased due to its delicate surface.
100–200	In the case of the intermediate films, the defect increased, but more slowly than in the case of thin and thick films.
≥250	In the case of thick films, the defect increased easily due to their greater brittleness/fragility.

**Table 3:** Electrical specifications of tested SunPower Maxeon Ne3 solar cells.

Data	Manufacturer Data (Non-Laminated)	Non-Laminated	Laminated (100 μm Film)
V_oc_ (V)	>0.731	0.733	0.726
I_sc_ (A)	>6.382	6.330	6.061
V_mp_ (V)	>0.625	0.627	0.624
I_mp_ (A)	>6.050	5.92	5.747
P_mpp_ (Wp)	>3.77	3.71	3.589
Fill Factor (%)	80.8	80.8	81
Efficiency (%)	>24.34	24.29	23.33

**Table 4:** Specification of tested SunPower Maxeon Ne3 solar cells.

Data	Non-Laminated			Laminated (100 μm Film)		
	Min	Max	RSD (%)	Min	Max	RSD (%)
V_oc_ (V)	0.728	0.738	0.77	0.725	0.731	1.81
I_sc_ (A)	6.109	6.33	0.28	6.032	6.421	0.24
V_mp_ (V)	0.616	0.639	1.04	0.604	0.629	1.47
I_mp_ (A)	5.793	6.039	0.79	5.724	6.05	1.15
P_mpp_ (Wp)	3.619	3.82	1.32	3.51	3.7	1.27
Fill Factor (%)	79.9	84.2	1.23	77.1	82.2	1.9
Efficiency (%)	24	24.77	1.12	23.01	23.79	0.85

**Table 5:** MPPT data of tested solar cell.

**Non-Laminated**				
**Irradiation (W/m^2^)**	**Voltage (V)**	**Current (A)**	**Power (W)**	**Fill Factor (%)**
1000	0.627	5.92	3.709	80.8
750	0.626	4.439	2.779	59.9
500	0.622	2.961	1.842	39.7
250	0.611	1.482	0.906	19.5
**Laminated**				
**Irradiation (W/m^2^)**	**Voltage (V)**	**Current (A)**	**Power (W)**	**Fill Factor (%)**
1000	0.624	5.748	3.588	81
750	0.622	4.317	2.684	61
500	0.618	2.872	1.776	40
250	0.606	1.437	0.871	19.8

**Table 6:** MPPT data for tested solar cells in different temperatures.

**Non-Laminated**				
**Temperature**	**Voltage (V)**	**Current (A)**	**Power (W)**	**Fill Factor (%)**
0	0.673	5.876	3.952	85.1
25	0.627	5.92	3.709	80.8
50	0.58	5.964	3.458	74.5
75	0.534	5.992	3.201	69
**Laminated**				
**Temperature**	**Voltage (V)**	**Current (A)**	**Power (W)**	**Fill Factor (%)**
0	0.671	5.706	3.827	87
25	0.624	5.748	3.588	81
50	0.578	5.779	3.343	76
75	0.532	5.813	3.092	70.2

## Data Availability

Not applicable.

## Abbreviations

AM	Air mass
EVA	Ethylene–vinyl acetate
GaAs	Gallium arsenide
I-V	Current–voltage
IR	Infrared
P-V	Power–voltage
PV	Photovoltaics
PVC	Polyvinyl chloride
RSD	Relative standard deviation
STC	Standard test conditions
TS	TwinStratos
UAV	Unmanned aerial vehicle
UV	Ultraviolet
